# Intakes of major food groups in China and UK: results from 100,000 adults in the China Kadoorie biobank and UK biobank

**DOI:** 10.1007/s00394-022-03031-6

**Published:** 2022-10-22

**Authors:** Keren Papier, Maria G. Kakkoura, Yu Guo, Anika Knuppel, Pei Pei, Tammy Y. N. Tong, Canqing Yu, Aurora Perez-Cornago, Wing Ching Chang, Junshi Chen, Jun Lv, Liming Li, Zhengming Chen, Huaidong Du, Timothy J. Key

**Affiliations:** 1 Cancer Epidemiology Unit, Nuffield Department of Population Health, University of Oxford, Oxford, UK; 2 Clinical Trial Service Unit and Epidemiological Studies Unit, Nuffield Department of Population Health, University of Oxford, Oxford, UK; 3 Chinese Academy of Medical Sciences, Beijing, China; 4 Fuwai Hospital Chinese Academy of Medical Sciences, Beijing, China; 5 MRC Unit for Lifelong Health and Ageing at UCL, Institute of Cardiovascular Science, University College London, London, UK; 6 Department of Epidemiology and Biostatistics, School of Public Health, Peking University Health Science Center, Beijing, China; 7 China National Center for Food Safety Risk Assessment, Beijing, China; 8 Center for Public Health and Epidemic Preparedness and Response, Peking University, Beijing, 100191 China; 9 Medical Research Council Population Health Research Unit, Nuffield Department of Population Health, University of Oxford, Oxford, UK

**Keywords:** China Kadoorie Biobank, UK Biobank, Dietary intake, Food groups, Cohort studies

## Abstract

**Purpose:**

Different populations may exhibit differences in dietary intakes, which may result in heterogeneities in diet–disease associations. We compared intakes of major food groups overall, by sex, and by socio-economic status (SES) (defined as both education and income), between participants in the China Kadoorie Biobank (CKB) and the UK Biobank (UKB).

**Methods:**

Data were from ~ 25,000 CKB participants who completed a validated interviewer-administered computer-based questionnaire (2013–2014) and ~ 74,000 UKB participants who completed ≥ 3 web-based 24-h dietary assessments (2009–2012). Intakes of 12 major food groups and five beverages were harmonized and compared between the cohorts overall, by sex and by SES. Multivariable-adjusted linear regression examined the associations between dietary intakes and body mass index (BMI) in each cohort.

**Results:**

CKB participants reported consuming more rice, eggs, vegetables, soya products, and less wheat, other staple foods (other than rice and wheat), fish, poultry, all dairy products, fruit, and beverages compared to UKB participants. Red meat intake was similar in both cohorts. Having a higher SES was generally associated with a higher consumption of foods and beverages in CKB, whereas in UKB dietary intakes differed more by education and income, with a positive association observed for meat and income in both UKB and CKB but an inverse association observed for education in UKB. Associations of dietary intakes with BMI varied between the two cohorts.

**Conclusion:**

The large differences in dietary intakes and their associations with SES and BMI could provide insight into the interpretation of potentially different diet–disease associations between CKB and UKB.

**Supplementary Information:**

The online version contains supplementary material available at 10.1007/s00394-022-03031-6.

## Introduction

Diet plays a key role in the aetiology of major chronic diseases such as cardiovascular disease (CVD) and diabetes, although some associations have been observed to differ across populations raising questions about the role of food intake habits in diet–disease associations. For instance, there is consistent evidence from North American and European adults that high red and processed meat intake is associated with a higher risk of ischaemic heart disease, but this association has not been observed in Asian populations [1]. Likewise, in large observational studies fresh fruit intake has been found to be associated with a ~ 30% lower risk of ischaemic heart disease in Asian adults [2], but only a ~ 10% lower risk in North American and European adults [3].

Differences in diet and disease associations between populations might relate to differences in dietary intakes and their socio-economic correlates. For instance, the consumption of red and processed meat has historically been lower in low- and middle-income countries (e.g. China) compared with high-income countries (e.g. UK) [4], and has only been increasing in recent years [5]. Therefore, it is possible that modest associations have not been observed due to low intakes. Heterogeneity in diet and disease associations might also relate to differences in nutritional status (e.g. body mass index (BMI)) between different populations. For example, the prevalence of overweight and obesity, a major risk factor for several diseases including CVD, is much higher in the UK compared with China (~ 60% versus ~ 30% based on a body mass index of 25 kg/m^2^ or more) [6, 7].

The China Kadoorie Biobank (CKB) and the UK Biobank (UKB) studies are two of the largest prospective cohort studies with information on usual dietary intakes in men and women living across China and the UK, respectively. Information on how dietary intakes compare across these two cohorts could help in the interpretation of differences in diet and disease associations, and could inform future research across different populations. Therefore, we compared dietary intakes in CKB and UKB overall, by sex and by socio-economic status (SES) (defined as both education and income), and investigated associations of dietary intake with BMI in each of the two cohorts.

## Methods

### Study population and data collection

Details of the study design, survey methods and population characteristics of the CKB and the UKB studies have been previously described elsewhere [8, 9]. In brief, the CKB is a population-based prospective study of over 0.5 million adults from ten geographically diverse regions (five urban and five rural) in China. At baseline (2004–2008), all permanent residents (aged between 30 and 79 years) living in pre-selected villages or urban communities were invited to participate in the study. About one in three responded and were enrolled in the study. During recruitment, participants completed a laptop-based questionnaire (collecting information including sociodemographic characteristics, medical history and lifestyle factors), and provided anthropometric measurements and biological samples. After completion of the baseline survey, two resurveys were undertaken in 5–6% of randomly selected surviving participants. In the present study, we used dietary data collected from ~ 25,000 participants during the second resurvey (2013–2014), since more detailed information on food consumption amounts was available.

The UK Biobank is a population-based prospective cohort study of middle-aged adults living in the UK. Between 2006 and 2010, 9.2 million adults registered with the National Health Service were invited to participate, of which ~ 5% (aged between 40 and 69 years) accepted the invitation and formed the baseline cohort. At the baseline assessment centre, a touchscreen questionnaire covering information on sociodemographic characteristics, medical history and lifestyle factors was administered and anthropometric and biological measurements were taken. In addition to the touchscreen questionnaire, participants who were recruited in 2009 and those who provided the UK Biobank with an email address were also invited to complete the Oxford WebQ (a self-administered web-based 24 h dietary questionnaire) on up to five separate occasions. The first occasion was in 2009 and the other four were during the follow up period (from February to April 2011, June to August 2011, October to December 2011 and April to June 2012). To account for variation in daily intakes and capture habitual intakes over the different seasons, we averaged daily intakes in participants who completed three or more Oxford WebQs. In total we used dietary data collected from ~ 74,000 participants who completed the Oxford WebQs around a similar time period to that of the CKB second resurvey (i.e. 2009–2012).

### Assessment of dietary intake and data harmonisation

In the CKB, information on the consumption of 12 major food groups and five non-alcoholic beverages (Supplementary Table S1) over the past 12 months was obtained using a validated interviewer-administered laptop-based questionnaire [10]. Participants were asked to report how often they consumed these food and beverage groups (5 categories: daily, 4–6 days/week, 1–3 days/week, monthly and never/rarely) and, except for non-consumers, how much they consumed, allowing us to estimate the average daily amount (in g or ml) of foods and drinks consumed. In addition, the amount of pure alcohol in g per session was calculated based on the type of alcoholic beverage and amount drunk on a typical day of drinking. This information was available for 293 female and 2734 male regular alcohol drinkers (at least once a week consumption), whereas intake of alcohol amount for the never regular, ex-regular and occasional drinkers was assumed to be zero. More information on the assessment of alcohol consumption in CKB has been reported previously [11]. In addition to these, consumption frequency of tea and coffee over the past 12 months was also collected using a validated interviewer-administered laptop-based questionnaire. All CKB participants were asked to report how often they drank tea (5 categories: at least once a week, every month but less than weekly, only at certain seasons, only occasionally, and never/almost never) and coffee (4 categories at least once a week, every month but less than weekly, only occasionally, and never/almost never) (Supplementary Table S1). Moreover, the CKB participants who consumed tea at least once a week (*n* = 7160) were further asked about days drinking in a typical week (1 to 2 or 3 to 5 days, or almost every day), cups (in 300 mL size) of tea consumed on a drinking day and amount (in grams) of tea leaves added each time, times of changing tea leaves on a drinking day, types of tea most commonly consumed (green tea, oolong tea, black tea, or others), and age when they started consuming tea weekly. However, for the purposes of this study, we only used the information on consumption frequency, which was available for all CKB participants in this study.

The validated Oxford WebQ dietary assessment tool used in UKB included questions on the consumption of 206 types of foods and 32 types of drinks [12]. Participants were asked to report how frequently they consumed these items in the past 24 h and the portions they consumed. For tea and coffee, participants were asked whether or not they had consumed any in the previous 24 h. Using this information, we calculated the mean daily intakes (in g or ml) for each food and beverage item [13, 14] and assigned regular tea and coffee drinking to those participants having responded yes to drinking tea or coffee in at least one of the Oxford WebQs. UKB participants with missing values for any of the 12 foods, five drinks, or alcohol were assigned a zero intake if their total energy intakes were within the plausible values (see exclusion criteria below). To facilitate the comparison with CKB data, we grouped food items together according to the definitions used in the CKB for the 12 major foods groups, five soft drinks, and alcohol. We did this by combining mean intakes of the relevant UKB food items that formed the CKB food groups. For instance to match the CKB wheat food group, we totalled all of the wheat items collected in the UKB WebQ to get a total wheat group (see Supplementary Table S1 for combined foods). All of the WebQ intakes were averaged across at least three Oxford WebQs to help determine habitual intakes.

### Exclusion criteria

A total of 25,239 CKB participants completed the second resurvey. A total of 78,737 UKB participants had completed three or more Oxford WebQs. Of these, the following participants were excluded from the current analysis: CKB participants with missing values of food intakes and BMI (*n* = 199); UKB participants who had reported energy intakes outside the range of 2093 (500 kcal) to 14,654 kJ (3500 kcal) for women and 3349 (800 kcal) to 16,747 kJ (4000 kcal) for men [15]; and UKB participants who reported an atypical dietary intake in the past 24 h due to illness or fasting (*n* = 4560). The analysis sample included 25,040 adults from CKB and 74,177 adults from UKB (Supplementary Tables S2–3).

### Statistical analysis

Sociodemographic, lifestyle, and health characteristics as well as mean dietary intakes (g/day or ml/day) were compared between the two cohorts. The characteristics compared included: age in years [mean (SD) and categories < 45, 45–49, 50–54, 55–59, 60–64, and 65 or over], education level [low (primary school/no formal education in CKB and national examination [16 yr] for UKB), medium [middle school or high school education in CKB and national examination (17/18 yr) for UKB], and high [college or university for both studies]), and annual household income [low (< 20,000 yuan/yr in CKB and < 18,000 £/yr in UKB), medium (20,000–49,999 yuan/yr in CKB and 18,000–< 52,000 £/yr in UKB), and high (≥ 50,000 yuan/yr in CKB and ≥ 52,000 £/yr in UKB)]. The levels for education and income were reported previously [16–18], although were further collapsed into three categories to create approximately equal-sized groups. We also compared smoking (never, ex, or current smoker), alcohol (never, ex or current drinker), BMI [mean (SD) and < 18.5, 18.5–24.9, 25.0–29.9, and 30 or more kg/m^2^), self-rated health status (excellent, good, fair, and poor), and region of residence (10 relevant for each cohort] (see Table 1 footnotes, and Supplementary Tables S2-4 for additional details). We also calculated adjusted mean dietary intakes by linear regression and investigated heterogeneity in dietary intakes by sex and by SES (defined as relating to both education and income levels), using fixed-effects meta-analysis between the two cohorts (presented as a phet) and independent Student’s t-tests within each cohort (presented as a p). Adjusted mean dietary intakes by regions in each cohort study were also calculated. We finally assessed the sex-specific association between dietary intake and BMI using multivariable-adjusted linear regression (adjusted for age, region, income level, education level, smoking, physical activity, and alcohol intake) in each cohort (*P* trend values are reported in the text). Analyses were carried out using the statistical package Stata (Stata Corp. College Station, TX, USA) version 16.1.

**Table 1:** Characteristics of the two cohort studies (analysis sample)

Characteristics	CKB *N* = 25,040	UKB^a^ *N* = 74,177
Time of data collection	2013–2014	2009–2012
Age (years), mean (SD)	59.5 (10.2)	56.4 (7.7)
Age (years), %		
< 45	6.8	9.4
45–49	15.2	12.6
50–54	13.6	15.9
55–59	17.3	20.3
60–64	17.6	26.0
≥ 65	29.6	15.8
Women, %	61.8	56.2
Education, %^b^		
Low	52.4	14.1
Medium	41.9	6.8
High	5.7	79.1
Annual household income, %^c^		
Low	21.0	13.9
Medium	36.0	53.0
High	43.1	33.2
Smoking, %^d^		
Never	68.2	57.7
Ex	6.4	35.9
Current	25.3	6.4
Alcohol, %^e^		
Never	64.0	5.8
Ex	2.9	–
Current	33.2	94.3
BMI (kg/m^2^), mean (SD)	24.2 (3.5)	26.6 (4.5)
BMI (kg/m^2^), %		
< 18.5	3.8	0.7
18.5–24.9	57.1	40.9
25.0–29.9	33.5	40.1
≥ 30	5.6	18.4
Self-rated health status, %		
Excellent	22.7	22.6
Good	21.1	60.0
Fair	43.4	15.3
Poor	12.9	2.5

^a^Proportion of participants with missing values ranges from 0.2% (health status) to 8.9% (income) in UKB. However, there are no missing values in CKB

^b^Low level of education indicates primary school/no formal education in CKB and national examination (16 yr) for UKB. Medium level of education represents middle school or high school education in CKB and national examination (17/18 yr) for UKB. High level of education means college or university for both studies

^**c**^Low level of income indicates < 20,000 yuan/yr in CKB and < 18,000 £/yr in UKB. Medium level of income indicates 20,000–49,999 yuan/yr in CKB and 18,000- < 52,000 £/yr in UKB. High level of income indicates ≥ 50,000 yuan/yr in CKB and ≥ 52,000 £/yr in UKB

^d^Within the CKB sample there were 22.6%, 15.8% and 61.6% men and 96.5%, 0.6% and 2.9% women, who were never smokers, ex-smokers, and current smokers, respectively. In the UKB sample, there were 52.5%, 39.7% and 7.8% men and 61.8%, 32.9% and 5.4% women, who were never smokers, ex-smokers, and current smokers, respectively

^e^Within the CKB sample there were 34.9%, 6.3% and 58.8% men and 81.9%, 0.7% and 17.3% women, who were never alcohol drinkers, ex-alcohol drinkers, and current alcohol drinkers, respectively. Current alcohol drinkers in CKB include the categories of occasional drinker (2261 men, 2208 women), monthly drinker (264 men, 94 women), reduced intake drinker (369 men, 82 women) and regular drinker (at least once per week; 2734 men, 293 women). Within the UKB sample, there were 4.7% and 95.4% men and 6.6% and 93.4% women, who were never alcohol drinkers and current alcohol drinkers, respectively

## Results

### Characteristics

Table 1 shows the population characteristics of CKB and UKB participants. In both cohorts, the mean (SD) age of participants was over 50 years (CKB 59.5 [10.2], UKB 56.4 [7.7]), and the majority of participants were women (61.8% and 56.2% respectively). The mean (SD) BMI was 24.2 (3.5) in CKB and 26.6 (4.5) kg/m^2^ in UKB. The levels of education, annual household income, alcohol consumption, smoking, and self-rated health status were somewhat different between the two cohorts. In CKB, the majority of participants had a low level of attained education (low 52.4% vs. high 5.7%), and had rated their health status as fair or poor (fair or poor 56.3% vs. good or excellent 43.8%). In terms of smoking and alcohol consumption status, the majority of men were current smokers (61.6%) and current drinkers (58.8%) whereas the majority of women were never smokers (96.5%) and never alcohol drinkers (81.9%) (See footnotes of Table 1). Conversely, in UKB, the majority of participants had a high level of attained education (low 14.1% vs. high 79.1%), currently consumed alcohol (never 5.8% vs. current 94.3%), never smoked (never 57.7% vs. current 6.4%), and had rated their health status as good or excellent (fair or poor 17.8% vs. good or excellent 82.6%). We observed similar characteristics in participants who were not eligible for the present study (Supplementary Table S5).

### Differences in dietary intakes overall

**Fig. 1:**
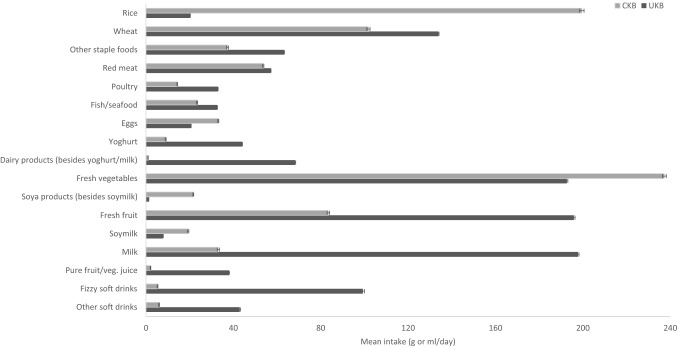
Mean intake (g/day or ml/day) of major food groups and drinks in CKB and UKB

Mean intakes of major food groups and drinks in the two cohorts are shown in Fig. 1. For staple foods, the mean intake of wheat and other staple foods (i.e. staple foods other than rice and wheat products) was lower, whereas the mean rice intake was nearly tenfold higher in CKB participants compared with UKB participants (200 vs. 20 g/day). For animal-sourced foods, CKB participants reported consuming less fish (23 vs. 33 g/day) and poultry (14 vs. 33 g/day) than UKB participants, but red meat intake was similar in the two cohorts (54 g/day CKB vs. 57 g/day UKB). The average intakes of yoghurt and other dairy products (i.e. besides yoghurt and milk, such as cheese) were over four times higher, while the mean intake of milk was sixfold higher in UKB. Conversely, egg consumption was over 1.5 times higher in CKB. The average intake of fresh vegetables (237 vs. 193 g/day) was higher in CKB than in UKB, and intake of soya products was much higher (22 vs. 1 g/day). In contrast, the average intake of fresh fruit was less than half in CKB than in UKB (84 vs. 196 g/day). The mean consumption of soymilk was ~ 2.5-fold higher, but the consumption of other beverages, including regular tea and coffee drinking was lower in CKB. For instance, the mean intakes of pure fruit/vegetable juice, fizzy soft and other soft drinks were 18-fold, ~ 19-fold and sevenfold higher in UKB than in CKB, respectively. Mean consumption levels of certain animal-sourced foods (red meat, poultry, fish and yoghurt) as well as fresh fruit in the urban study areas of CKB were also fairly similar to those observed in UKB (Supplementary Table S4).

### Differences in dietary intakes by sex and SES

Dietary intakes by sex in each cohort are shown in Table 2. Overall, the sex-specific differences in dietary intakes between the two cohorts were similar to those observed in Fig. 1. For both men and women, there were significant differences (phet < 0.0001) between CKB and UKB for all foods except for red meat in men (phet = 0.12). Within CKB, dietary intakes of most items were significantly different between the two sexes (*P* < 0.0001), except for the intake of other staple foods, other dairy products (besides yoghurt and milk), milk, soymilk, pure fruit/vegetable juice and coffee. Particularly, intakes of yoghurt and fresh fruit were significantly higher in women, while intakes of the remaining items were significantly higher in men. Within UKB, except for other dairy products (besides yoghurt and milk) the intakes of all other major foods and beverages differed between men and women (*P* < 0.01), with men consuming more wheat, red meat, milk, fizzy soft drinks, other soft drinks, and alcohol and women consuming much more fresh vegetables, and fresh fruit.

**Table 2:** Dietary intake^a^ by sex in CKB and UKB participants

Food groups and drinks, mean g/day or mean ml/day	CKB		UKB	
	Women *N* = 15,462	Men *N* = 9578	Women *N* = 41,668	Men *N* = 32,509
Staple foods (g/day)				
Rice	181.7^d,e^	228.3^d,e^	19.4^d,e^	21.2^d,e^
Wheat	90.5^d,e^	120.1^d,e^	118.5^d,e^	153.6^d,e^
Other staple foods	37.4^d^	37.2^d^	65.1^d,e^	60.7^d,e^
Animal-sourced foods (g/day)				
Red meat	46.4^d,e^	65.4^e^	50.0^d,e^	66.2^e^
Poultry	12.2^d,e^	17.8^d,e^	32.1^d,e^	34.0^d,e^
Fish/seafood	20.5^d,e^	28.0^d,e^	33.0^d,e^	32.1^d,e^
Eggs	31.2^d,e^	36.2^d,e^	20.1^d,e^	21.2^d,e^
Yoghurt	10.1^d,e^	7.2^d,e^	50.7^d,e^	35.3^d,e^
Dairy products other than milk and yoghurt^b^	1.0^d^	0.9^d^	68.5^d^	68.0^d^
Plant-based foods (g/day)				
Fresh vegetables	231.5^d,e^	246.6^d,e^	213.4^d,e^	166.1^d,e^
Soya products other than soymilk	19.9^d,e^	24.5^d,e^	1.6^d,e^	1.1^d,e^
Fresh fruit	87.4^d,e^	77.1^d,e^	205.3^d,e^	183.8^d,e^
Drinks				
Soymilk (ml/day)	19.0^d^	19.8^d^	9.9^d,e^	5.2^d,e^
Milk (ml/day)	33.8^d^	32.1^d^	191.2^d,e^	206.2^d,e^
Pure fruit/veg. juice (ml/day)	2.1^d^	2.0^d^	37.4^d,e^	38.9^d,e^
Fizzy soft drinks (ml/day)	3.4^d,e^	8.3^d,e^	99.7^d,e^	118.8^d,e^
Other soft drinks (ml/day)	4.7^d,e^	8.0^d,e^	96.1^d,e^	103.5^d,e^
Alcohol (g/day)	0.4^d,e^	18.9^d,e^	12.0^d,e^	21.4^d,e^
Tea (% of regular drinkers)^c^	17.6^d,e^	46.3^d,e^	88.2^d,e^	86.0^d,e^
Coffee (% of regular drinkers)^c^	1.3^d^	1.3^d^	81.8^d,e^	84.3^d,e^

^a^Adjusted for age (< 45, 45–49, 50–54, 55–59, 60–64, and ≥ 65 years) and region (10 areas) in each cohort

^b^These products include cheese, milk powder, dairy-based smoothies, drinks or desserts

^c^Regular drinking indicates consumption of at least once a week in CKB and reported on at least one WebQ in UKB. Percentage values presented are crude, i.e. not adjusted for age and region

^d^Phet < 0.05 for fixed-effects meta-analysis between CKB and UKB men and CKB and UKB women

^e^*P* < 0.05 for independent Student’s *T* test between CKB men and women and UKB men and women

**Fig. 2:**
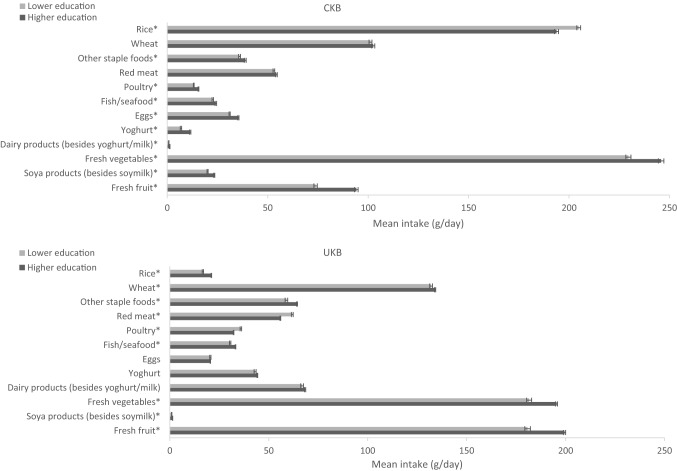
Adjusted^a^ mean intake (g/day) of food groups in participants with lower and higher levels (medium and high combined) of education in CKB and UKB. ^a^Adjusted for age (< 45, 45–49, 50–54, 55–59, 60–64, and ≥ 65 years), sex (men and women), region (10 relevant for each cohort) and income (lower [low and medium combined] and higher levels). More details on the definitions of lower and higher levels of education in each study can be found in Table 1. In CKB the mean intake of dairy products (besides yoghurt and milk) was 0.8 and 1.3 g/day in the lower and higher education groups, respectively; in UKB the mean intake of soya products was 1.0 and 1.4 g/day in the lower and higher education groups, respectively. Phet < 0.01 for fixed-effects meta-analysis between CKB and UKB low education levels and CKB and UKB high education levels. *P < 0.05 for independent Student’s *T* test between CKB low and high education and UKB low and high education

**Fig. 3:**
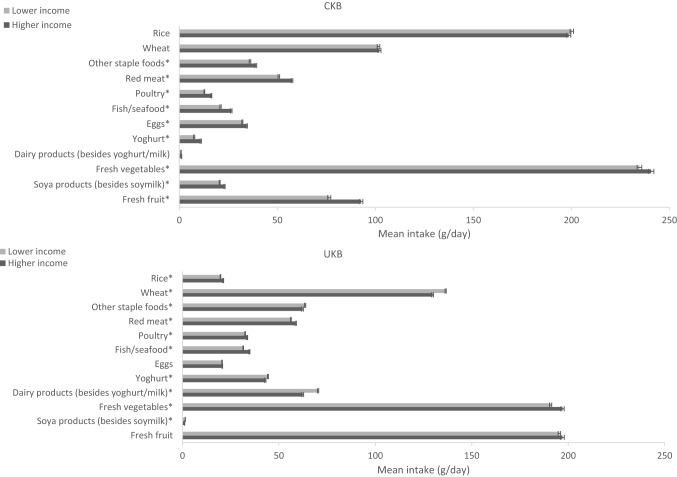
Adjusted^a^ mean intake (g/day) of food groups in participants with lower (low and medium combined) and higher levels of household income in CKB and UKB. ^a^Adjusted for age (< 45, 45–49, 50–54, 55–59, 60–64, and ≥ 65 years), sex (men and women), region (10 relevant for each cohort) and education (lower and higher levels [medium and high combined]). More details on the definitions of lower and higher levels of household income in each study can be found in Table 1. In CKB the mean intake of dairy products (besides yoghurt and milk) was 0.9 and 1.1 g/day in the lower and higher income groups, respectively; in UKB the mean intake of soya products was 1.5 and 1.1 g/day in the lower and higher income groups, respectively. Phet < 0.01 for fixed-effects meta-analysis between CKB and UKB low income levels and CKB and UKB high-income levels. *P < 0.05 for independent Student’s *T* test between CKB low and high income and UKB low and high income

Major food and beverage intakes by SES in the two cohorts are shown in Figs. 2, 3 and Supplementary Fig. S1-2, respectively. Significant differences between the two cohorts were observed among both low and high SES participants for all foods (phet < 0.01). When comparing the consumption levels in high and low SES groups within each cohort, CKB participants with a higher SES generally reported consuming significantly more foods and drinks than those with a lower SES (*P* < 0.05 for other staple foods, poultry, fish/seafood, eggs, yoghurt, fresh vegetables, soya products, fresh fruit, soymilk, milk, and pure fruit/vegetable juice). Particularly, differences were apparent for fresh vegetables (low income 235 g/day vs. high income 241 g/day; low education 230 g/day vs. high education 246 g/day) and fresh fruit (low income 77 g/day vs. high income 93 g/day; low education 74 g/day vs. high education 94 g/day). Participants with a higher education level reported consuming a lower amount of rice and a slightly higher amount of other dairy products than those with a lower education (*P* < 0.001). For income, participants with a higher income level reported consuming more red meat (*P* < 0.001) and other soft drinks than those with a lower income (*P* < 0.01). For alcohol, the consumption was slightly but significantly higher among participants with a higher income level (*P* < 0.01). In UKB, intakes of rice, fish, and fresh vegetables were higher, while intakes of fizzy drinks and other soft drinks were lower in adults with a higher SES (*P* < 0.001). Furthermore, participants with a higher education reported consuming more wheat, other staple foods, less red meat, less poultry, and more fresh fruit, pure fruit/vegetable juice, and slightly more soya products (other than soymilk) than those with a lower education (*P* < 0.001). Finally, participants with a higher income reported consuming more red meat, poultry, alcohol, and slightly less wheat, other staple foods, dairy products, and soya products (including soymilk) than participants with a lower income (*P* < 0.01).

### Differences in BMI by dietary intakes

**Fig. 4:**
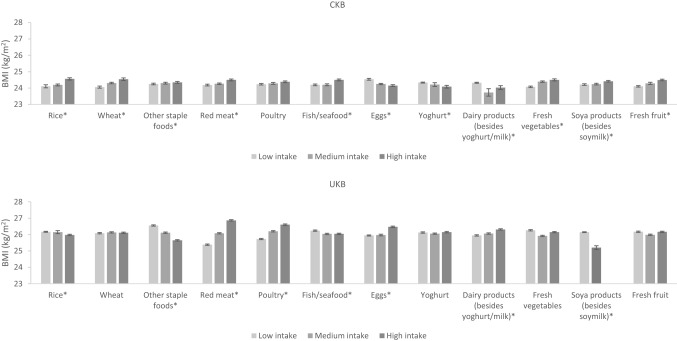
Adjusted^a^ mean BMI by intake of food groups in CKB and UKB in women. ^a^Adjusted for age (< 45, 45–49, 50–54, 55–59, 60–64, and ≥ 65 years), region (10 relevant for each cohort), education level (low, medium, high), income level (low, medium, high), smoking (never, ex-smoker, current smoker), physical activity (quartiles of metabolic equivalent of task hours per day in CKB and quartiles of metabolic equivalent of task hours per week in UKB), and alcohol (never, ex-drinker, current drinker in CKB and never, current drinker in UKB). In CKB intake of food groups was divided into tertiles where possible or low, medium and high intake corresponding to never/rarely, monthly and weekly intake, respectively. In UKB intake of food groups was divided into tertiles where possible or ‘none’ versus ‘any’ where intakes were too low. *P trend < 0.05 across the intake groups

**Fig. 5:**
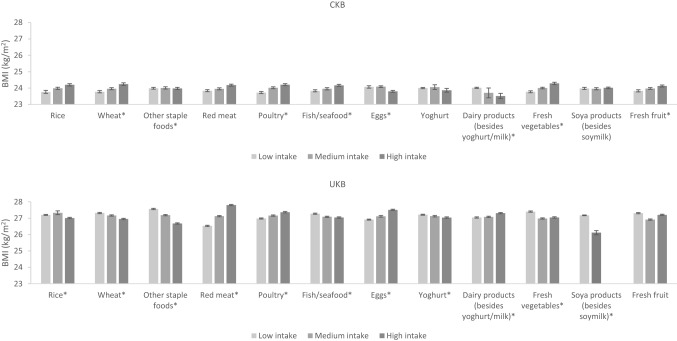
Adjusted^a^ mean BMI by intake of food groups in CKB and UKB in men. ^a^Adjusted for age (< 45, 45–49, 50–54, 55–59, 60–64, and ≥ 65 years), region (10 relevant for each cohort), education level (low, medium, high), income level (low, medium, high), smoking (never, ex-smoker, current smoker), physical activity (quartiles of metabolic equivalent of task hours per day in CKB and quartiles of metabolic equivalent of task hours per week in UKB), and alcohol (never, ex-drinker, current drinker in CKB and never, current drinker in UKB). In CKB intake of food groups was divided into tertiles where possible or low, medium and high intake corresponding to never/rarely, monthly and weekly intake, respectively. In UKB intake of food groups was divided into tertiles where possible or ‘none’ versus ‘any’ where intakes were too low. *P trend < 0.05 across the intake groups

Differences in BMI by dietary intakes in men and women in both cohorts are shown in Fig. 4, 5 and Supplementary Fig. S3-4. In women, the associations of staple foods with BMI were in the opposite direction in CKB and UKB; CKB women with higher intake of rice and other staple foods had a higher BMI while UKB women with higher intake had a lower BMI. For animal-sourced foods, higher intake of red meat and poultry was associated with a higher BMI (although the *P* trend for poultry was not statistically significant for CKB women), while higher milk intake was associated with a lower BMI in women of both cohorts. Of particular note, we observed that UKB women who had the highest intake of red meat had a 1.5 kg/m^2^ higher BMI than women who had the lowest intake of red meat. Associations for fish and eggs were dissimilar; CKB women with higher fish consumption and lower egg consumption had a higher BMI, while the opposite associations were observed in UKB women. For plant-based non-staple foods, CKB women with higher intakes of fresh vegetables and soya products had a slightly higher BMI, while the opposite was observed in UKB women (although the *P* trend for fresh vegetables was not statistically significant for UKB women). For beverages, women with a higher consumption of fizzy drinks and other soft drinks had a higher BMI in both cohorts (although the *P* trend for fizzy drinks was not statistically significant for CKB women). Notably, UKB women who had the highest consumption of fizzy drinks had a 2 kg/m^2^ higher BMI than women who had the lowest consumption of fizzy drinks. In CKB, women with higher tea intake had higher BMI, while in UKB the opposite was observed.

In men, associations between dietary intake of several foods and BMI were similar to those observed in women including for red meat, poultry, milk, fresh vegetables, fizzy drinks, and other soft drinks (although the *P* trend values for red meat, milk, fizzy drinks, and other soft drinks were not statistically significant for CKB men). Similarly, but to a lesser degree than the women, UKB men who had the highest intake of fizzy drinks had a 1.6 kg/m^2^ higher BMI than men with the lowest intake. Men with higher consumption of other staple foods had lower BMIs in both cohorts (UKB men in particular). Higher wheat intake was also associated with a higher BMI in CKB men, while the opposite was observed in UKB men. CKB men who had higher fish intake but lower intake of “other dairy products” (besides yoghurt and milk) had a higher BMI, while the opposite associations were observed in UKB men. For drinks, a higher intake of pure fruit/vegetable juice was associated with a lower BMI, while higher alcohol intake was associated with a higher BMI among men of both cohorts, particularly in UKB men (*P* trend for alcohol was not statistically significant for CKB men). Higher tea intake was associated with a higher BMI in CKB men but with a lower BMI in UKB men. The *P* trend values for all the aforementioned associations were less than 0.05 unless otherwise specified, indicating that the associations between dietary intake and BMI are significant.

## Discussion

In this study, we compared mean intakes of major food groups from the CKB and UKB cohorts. CKB participants reported consuming less wheat products, other staple foods, poultry, fish, dairy products, fresh fruit and beverages other than soymilk, but more rice, eggs, fresh vegetables, soya products compared to UKB participants. Overall, having a higher SES was associated with a higher consumption of foods and beverages in CKB, whereas in UKB consumption patterns differed much more by education and income levels, with opposite associations observed for meat consumption between education and income in UKB, but not in CKB. We observed some similarities in the associations of food and beverages with BMI among the two cohorts (for instance adults who consumed more red meat, poultry, and soft drinks but less milk had a higher BMI) although most of the associations between diet and BMI were not similar among CKB and UKB (for example CKB adults with higher fish consumption had a higher BMI, while the opposite associations were observed in UKB adults).

Similar to our results, it has been previously shown that during the period of 2012–2016 rice has contributed a small proportion of the cereals purchased in the UK [19]. In China, staple food intake has traditionally been related to the region of residence, with more wheat typically consumed in the north and rice in the south, particularly in the areas near the Yangtze River [20]; the overall consumption of cereals has decreased in recent decades with a large decline in coarse grains due to advances in food processing technologies and a shift towards a greater consumption of animal foods [21].

It might be expected that UKB participants would consume higher amounts of all animal-sourced foods given that the UK is a high-income Western country, but this was not the case. Despite the much lower dairy consumption in CKB compared with UKB, which is consistent with the very low mean dairy intake levels still observed in China [5], the differences in fish and meat intakes were not as large, likely reflecting the large increases in the mean consumption of these in China over the last three decades [5, 21] and the consistently moderate consumption in the UK [22]. Of particular note, red meat intakes were very similar in the two cohorts. In the UK, red meat intake has steadily declined in recent years [22]. Conversely, the opposite has been observed in China, with intakes from red meat now surpassing all other meat types [5]. National data from 2010–2012 showed that intakes of pork, livestock meat and poultry were 64 g/day, 8 g/day and 24 g/day, respectively [21]. The mean egg intake in CKB was higher than in UKB, with the UKB data being similar to what was reported in the UK at a similar time (21 g/day) [4].

The observation of considerably lower fresh fruit intakes in CKB compared with UKB is in line with what has been observed in nationally representative surveys from China and the UK [5, 23, 24]. Although fruit intake in China has been steadily increasing over the past three decades [5], mean intakes remain much lower than the recommended dietary guidelines for Chinese (minimum of 200 g/day) [25]. On the other hand, vegetable intakes were substantially higher in CKB than in UKB. The differences in fruit and vegetable intakes between the two cohorts might partly reflect differences in diet culture; vegetables are considered a necessary ingredient in Chinese cuisine and fruit is usually consumed as a snack which is not a necessary component of regular meals [24]. Likewise, CKB participants consumed much more soy, which is a commonly used ingredient in Chinese cuisine [26] and its consumption is specifically encouraged in the Chinese dietary guidelines [25].

Sales and consumption of soft drink and pure fruit juice have been increasing steadily in China in recent years [5, 27, 28], but intakes remain lower than what has been observed in high-income countries, including in the UK. This is consistent with what we observed in the current analysis, even though soft drink intake in UKB was lower than that reported in the general UK population over the last decade [23]. UKB participants are on average more health conscious than the general UK population and are less socioeconomically deprived [9], which we found was associated with lower soft drink consumption. It is worth noting that soft drink consumption has changed in the UK in recent years, with higher intakes of low calorie soft drinks and lower intakes of sugar-sweetened beverages [23].

When comparing the dietary intakes by SES in the two cohorts, intakes of fish and fresh vegetables were higher in those with higher SES in both CKB and UKB. However, in CKB higher SES was also associated with a higher intake of most foods and beverages, while in UKB higher SES was additionally associated with lower intakes of fizzy drinks and other soft drinks. Moreover, while higher income levels were associated with higher meat intakes in both cohorts, though we observed larger differences in red meat intake levels when comparing participants with high to low income levels in CKB, higher education levels were associated with lower meat intakes in UKB. In line with our results, an analysis of meat consumption in relation to income from 120 countries showed that meat consumption increases with income at lower levels of income, but decreases at higher income levels (inverted *U* shaped curve of consumption) [29]. During the last few decades, China has experienced rapid economic development. Concomitant increases in income levels have been associated with a shift towards a ‘Western diet’ containing more processed and animal-sourced foods and fewer indigenous staple grains, legumes and vegetables [30]. This “nutrition transition” is thought to occur as countries enter the early stages of economic development. At later stages, negative health behaviours begin to reverse [31]; this is commonly observed in high-income countries where a higher SES is associated with a higher consumption of health-promoting foods (e.g. higher intakes of fruit and wholegrains and lower intakes of processed meat) [23, 29, 32–34].

Although a higher SES was associated with higher intakes of most foods and beverages in CKB, higher education was associated with lower rice intake, particularly in four study regions (Zhejiang, Haikou, Suzhou, Sichuan) where rice is the dominant staple food (data not shown). This finding is consistent with previous work by Chang et al*.* [35]*,* based on data from the China Health and Nutrition Survey (CHNS) (2006–2009), which found an increase in the consumption of diverse coarse staple foods (e.g. millet, sorghum and corn) but not rice in communities with a high SES, measured by indicators of income, education, and household characteristics, and found to be driven predominantly by having a higher level of attained education. These results might also relate to the development of efficient commodity transport systems and thus higher diversity of staple supply in local markets of areas with a higher SES [35].

Higher income was associated with higher alcohol intake in both CKB and UKB. These findings have also been demonstrated by national UK data [36, 37] and by previous studies among male alcohol drinkers in China [38, 39]. It is possible that high income reflects better affordability and economic access to food and drink, but does not necessarily reflect healthier behaviours [40, 41].

We observed some similarities for associations between dietary intakes and BMI between the cohorts, including that men and women with lower milk intakes had a higher BMI in both cohorts. This has also been reported in previous observational studies [42], but the opposite has been found in Mendelian randomization studies [43, 44], perhaps because the results from the observational analyses may have been influenced by residual confounding and/or reverse causality. Another similarity between the two cohorts is that participants who had higher intakes of fizzy drinks and other soft drinks had a higher BMI; this aligns with previous results from China and the UK [45, 46]. The final similarity we found between the two cohorts was that participants who ate more red meat and poultry had a higher BMI. Previous prospective cohort studies have also reported that higher meat intake is associated with weight gain in European [47] and North American [48] adults. However, as with the associations for milk, fizzy drinks, and other soft drinks, the associations between diet and BMI in the present study are cross-sectional and thus might reflect reverse causality and/or differences in energy intakes between participants with high or low consumption of these foods and beverages. Overall, the associations of diet with BMI largely differed between the two cohorts. BMI is an important risk factor for several non-communicable diseases (including e.g. CVD [49]), therefore the different associations between dietary intakes and BMI in these two cohorts might be relevant for some of the different diet–disease associations observed across different populations.

Strengths of the current study include the large sample size and the detailed information on habitual dietary intakes. However, this study is not without its limitations. Dietary intake was self-reported in both CKB and UKB, and therefore intakes may have been misreported. Nevertheless, the dietary assessment tools in both cohorts have been validated [10, 12]. Additionally, some important condiments and dietary factors related to disease burden [5] were not captured in the dietary assessment tools (e.g., the CKB questionnaire did not collect information on nut intake). Moreover, several foods (e.g. soya products) and drinks were only partially comparable across the two cohorts. However, the dietary data were harmonised between the two cohorts and cross-cohort comparability was maximised by matching the food and drink items available in each cohort. Another limitation was that energy intake could not be reliably calculated in the CKB and therefore was not accounted for in our analyses. Lastly, the cohorts do not fully represent their respective national populations, so the generalizability of our findings might be limited by the structure of the two cohort studies.

## Conclusions

We compared mean intakes of major food groups between CKB and UKB participants. Our results indicate that there are large differences in dietary intakes between these two cohorts, and that associations between diet with SES and between diet with BMI are not similar. These findings highlight the need to consider differences in dietary intakes (i.e. amounts and definitions) and their correlates when comparing diet and disease associations across different populations. Such information might help us interpret potentially different future associations between diet and disease risk in the two cohorts.

## Supplementary Information

Below is the link to the electronic supplementary material.

Supplementary file1 (PDF 653 KB)
