# Supplementary material for: Intakes of major food groups in China and UK: results from 100,000 adults in the China Kadoorie biobank and UK biobank
Source: Eur J Nutr. Author manuscript; Available in PMC 2023 Mar 1. (PMC9941233; doi:10.1007/s00394-022-03031-6)
Supplement: Supplementary file [file EMS162130-supplement-Supplementary_file.pdf]

Intakes of major food groups in China and UK: results from 100,000 adults in the China Kadoorie Biobank and UK Biobank

Supplementary Material

Table of Contents

Table S1. UKB WebQ food items or groups within each CKB food groups 2

Table S2: Dietary intake[a] by study region in CKB (N = 25,040) 3

Table S3: Dietary intake[a] by study region in UKB (N = 74,177) 4

Table S4: Dietary intake by UKB and by urban and rural areas in CKB 5

Table S5. Baseline characteristics of the non-analysed sample in each cohort 6

Figure S1: Adjusted[a] mean intake (ml or g/day) of drinks in participants with lower and higher levels (medium and high combined) of education in CKB and UKB 7

Figure S2: Adjusted[a] mean intake (ml or g/day) of drinks in participants with lower (low and medium combined) and higher levels of household income in CKB and UKB 8

Figure S3. Adjusted[a] mean BMI by intake of drinks in CKB and UKB in women 9

Figure S4. Adjusted[a] mean BMI by intake of drinks in CKB and UKB in men 10

Table S1. UKB WebQ food items or groups within each CKB food groups

CKB food groups in the 2nd resurvey	WebQ foods/or food groupings
**Staple foods**	
Rice	White rice, brown rice, sushi
Wheat	White bread, wholemeal bread, mixed/brown bread, savoury crackers (e.g. crispbread), biscuits, bran cereal, biscuit cereal, white pasta, wholemeal pasta, snack pot, couscous
Other staple foods (other grains except rice and wheat products)	Other bread, oat cereal (non sugar), oat cereal (sugar), muesli, other cereal (sugar), other grain intake
**Animal-sourced foods**	
Red Meat	Pork, beef, lamb, other meat, offal, processed meat
Poultry	Poultry, breaded/battered chicken
Fish/seafood	White fish and tinned tuna, shellfish, oily fish, breaded/battered fish
Eggs	Eggs, egg dishes
Yoghurt	Low and full fat yoghurt
Dairy products other than yoghurt and milk	High fat cheese, medium and low-fat cheese, cream, milk dairy desserts, milk-based and powdered drinks
**Plant-based foods**	
Fresh vegetables	Raw salad, green leafy/cabbages, root vegetables, tomatoes, allium vegetables, other vegetables (including mushrooms, fruiting and mixed vegetables), peas/sweetcorn, vegetable side dishes
Soya products other than soymilk	Meat substitutes (soy), soy desserts and yoghurt
Fresh fruit	Citrus, berries, apples and pears, other fruit (e.g. bananas)
**Drinks**	
Soymilk	Soy milk
Milk	Whole milk, semi skimmed and skimmed milk
Pure fruit/vegetable juice	Pure fruit/vegetable juice
Fizzy soft drinks	Fizzy drinks (low and high calorie)
Other soft drinks	Fruit drinks/squash cordial
Alcohol (beer, wine, rice wine, weak spirits [<40% alcohol], strong spirits [≥40% alcohol])	Red wine, white wine, rose, fortified wine, spirits, other alcohol, beer
Tea	Consumed tea in at least one WebQ
Coffee	Consumed coffee in at least one WebQ

Table S2: Dietary intake[a] by study region in CKB (N = 25,040)

Food groups and drinks, mean g/day or mean ml/day	CKB									
	Urban					Rural				
	Qingdao N = 1643	Harbin N = 2170	Haikou N = 1385	Suzhou N = 2827	Liuzhou N = 2781	Sichuan N = 2789	Gansu N = 2492	Henan N = 3102	Zhejiang N = 2940	Hunan N = 2911
**Staple foods (g/day)**										
**Rice**	49.5	142.3	221.5	338.3	193.6	295.2	26.9	42.1	367.6	241.7
**Wheat**	200.3	124.7	61.5	26.8	52.5	19.7	313.8	165.6	26.7	73.5
**Other staple foods**	37.6	40.5	17.5	23.3	34.5	6.3	18.3	161.1	8.2	4.2
**Animal-sourced foods (g/day)**										
**Red meat**	52.1	46.9	68.1	51.4	80.5	100.4	18.4	24.2	40.2	59.6
**Poultry**	11.0	13.6	27.3	15.0	33.0	11.9	10.5	4.9	14.7	7.3
**Fish/seafood**	53.2	21.1	87.6	44.4	30.1	7.5	1.7	0.9	10.9	21.1
**Eggs**	59.8	39.5	16.0	24.7	29.2	23.8	42.3	39.8	25.3	35.3
**Yoghurt**	24.2	27.7	5.2	6.4	14.4	4.9	7.4	1.0	4.3	4.2
**Dairy products other than milk and yoghurt[b]**	1.0	1.1	2.7	0.6	2.0	0.2	2.2	0.3	0.5	0.5
**Plant-based foods (g/day)**										
**Fresh vegetables**	341.6	254.6	224.9	288.9	258.9	249.8	239.2	218.7	170.5	174.5
**Soya products other than soymilk**	30.8	36.5	10.9	18.3	27.4	9.2	21.2	15.3	29.8	19.1
**Fresh fruit**	182.4	104.4	53.2	51.7	135.9	63.0	89.0	45.2	77.7	68.8
**Drinks**										
**Soymilk (ml/day)**	27.4	45.2	18.2	12.2	29.8	13.8	13.6	20.7	11.8	8.9
**Milk (ml/day)**	95.9	70.0	7.2	19.8	57.9	32.7	19.1	33.6	10.6	6.4
**Pure fruit/veg. juice (ml/day)**	2.4	4.8	2.5	1.3	6.1	0.0	2.1	0.3	1.3	1.2
**Fizzy soft drinks (ml/day)**	3.1	11.2	12.1	2.3	4.0	4.4	11.5	2.9	3.6	2.6
**Other soft drinks (ml/day)**	3.0	6.8	9.9	1.1	5.0	2.0	14.5	13.8	2.3	2.9
**Alcohol (g/day)**	8.9	8.7	6.3	9.6	6.1	12.1	3.2	9.3	6.4	3.9
**Tea (% of regular drinkers)[c]**	56.9	19.4	55.4	29.9	26.2	26.7	35.0	2.6	23.8	36.6
**Coffee (% of regular drinkers)[c]**	1.8	3.1	7.9	0.6	1.7	0.1	0.4	0.1	1.0	0.2

[a]Adjusted for age (<45, 45–49, 50–54, 55–59, 60–64, and ≥65 years) and sex.
[b]These products include cheese, milk powder, dairy-based smoothies, drinks or desserts.
[c]Regular drinking indicates consumption of at least once a week. Percentage values presented are crude, i.e. not adjusted for age and sex.

Table S3: Dietary intake[a] by study region in UKB (N = 74,177)

Food groups and drinks, mean g/day or mean ml/day	UKB									
	Scotland N = 3897	NE England N = 6813	Yorkshire N = 11,589	NW England N = 9002	E mid-lands N = 4438	W mid-lands N = 5896	Wales N = 2247	London N = 15,769	SE England N = 6797	SW England N = 7729
**Staple foods (g/day)**										
**Rice**	19.1	18.4	17.3	19.5	17.3	18.1	18.9	25.8	20.3	19.1
**Wheat**	144.8	137.0	134.3	135.8	132.9	134.1	131.7	132.1	129.5	131.5
**Other staple foods**	65.8	61.2	63.2	64.3	64.4	64.8	61.1	62.4	63.6	62.1
**Animal-sourced foods (g/day)**										
**Red meat**	54.3	58.8	57.9	58.8	56.6	58.1	58.5	54.6	58.0	57.3
**Poultry**	30.9	35.2	31.9	35.7	30.5	34.5	33.7	33.1	32.4	30.2
**Fish/seafood**	33.7	29.7	32.8	32.3	32.1	30.7	30.6	36.0	32.5	30.6
**Eggs**	20.2	21.5	20.7	20.6	20.6	19.8	19.8	20.8	20.0	20.8
**Yoghurt**	44.5	45.5	45.8	44.9	45.9	43.8	43.6	40.9	44.4	43.5
**Dairy products other than milk and yoghurt[b]**	67.1	67.1	67.1	66.8	73.5	73.2	69.6	65.2	70.1	71.3
**Plant-based foods (g/day)**										
**Fresh vegetables**	165.1	191.0	194.5	187.6	201.0	192.4	191.9	194.8	192.3	203.0
**Soya products other than soymilk**	1.1	1.0	1.2	1.3	1.2	1.3	1.2	1.7	1.4	1.5
**Fresh fruit**	200.7	194.4	197.5	196.7	193.8	196.3	199.7	195.0	192.2	196.1
**Drinks**										
**Soymilk (ml/day)**	6.3	6.4	7.6	7.2	7.9	7.7	7.7	8.5	8.3	9.4
**Milk (ml/day)**	189.6	202.1	205.7	206.7	211.3	203.8	202.1	181.1	198.9	195.4
**Pure fruit/veg. juice (ml/day)**	41.6	33.6	34.0	35.1	37.0	35.3	34.1	44.4	40.4	38.3
**Fizzy soft drinks (ml/day)**	105.4	115.3	100.8	104.1	108.1	112.6	116.3	84.5	93.8	89.4
**Other soft drinks (ml/day)**	37.7	43.7	42.4	44.8	51.4	48.7	42.1	38.1	42.1	45.1
**Alcohol (g/day)**	15.0	15.2	16.1	15.6	15.1	14.7	15.7	18.2	15.6	16.2
**Tea (% of regular drinkers)[c]**	85.5	85.7	87.0	86.8	87.0	86.8	85.1	88.6	87.6	88.7
**Coffee (% of regular drinkers)[c]**	82.9	82.4	83.4	81.9	83.2	81.2	82.4	83.0	84.4	83.5

[a]Adjusted for age (<45, 45–49, 50–54, 55–59, 60–64, and ≥65 years) and sex.
[b]These products include cheese, milk powder, dairy-based smoothies, drinks or desserts.
[c]Regular drinking indicates consumption of at least once a week. Percentage values presented are crude, i.e. not adjusted for age and sex.

Table S4: Dietary intake by UKB and by urban and rural areas in CKB

Food groups and drinks, mean g/day or mean ml/day	CKB		UKB
	Urban N = 10,806	Rural N = 14,234	N = 74,177
**Staple foods (g/day)**			
**Rice**	202.6	197.3	20.2
**Wheat**	84.0	115.3	133.9
**Other staple foods**	31.2	42.0	63.2
**Animal-sourced foods (g/day)**			
**Red meat**	60.2	48.7	57.1
**Poultry**	20.3	9.8	32.9
**Fish/seafood**	42.9	8.6	32.6
**Eggs**	33.0	33.2	20.6
**Yoghurt**	15.3	4.2	44.0
**Dairy products other than milk and yoghurt**[a]	1.4	0.7	68.3
**Plant-based foods (g/day)**			
**Fresh vegetables**	274.1	209.4	192.7
**Soya products other than soymilk**	25.2	18.9	1.3
**Fresh fruit**	103.8	68.0	195.9
**Drinks**			
**Soymilk (ml/day)**	26.4	13.8	7.8
**Milk (ml/day)**	49.6	20.6	197.8
**Pure fruit/veg. juice (ml/day)**	3.6	1.0	38.0
**Fizzy soft drinks (ml/day)**	5.9	4.8	99.3
**Other soft drinks (ml/day)**	4.7	7.0	42.9
**Alcohol (g/day)**	8.0	7.1	16.1
**Tea (% of regular drinkers)**[b]	34.2	24.3	87.3
**Coffee (% of regular drinkers)**[b]	2.5	0.4	82.9

[a]These products include cheese, milk powder, dairy-based smoothies, drinks or desserts.
[b]Regular drinking indicates consumption of at least once a week in CKB and reported on at least one WebQ in UKB. Percentage values presented are crude, i.e. not adjusted for age and sex.

Table S5. Baseline characteristics of the non-analysed sample in each cohort

Characteristics	CKB N = 25,040[a]	CKB N = 487,486[b]	UKB N = 428,311[c]
**Time of data collection**	2004 - 2008	2004 - 2008	2006 - 2010
**Age (years), mean (SD)**	51.5 (10.1)	52.0 (10.7)	56.5 (8.2)
**Age (years), %**			
<45	31.2	31.6	10.5
45-49	14.1	13.4	13.2
50-54	18.4	17.0	15.1
55-59	14.6	13.6	17.7
60-64	10.1	9.7	23.9
≥65	11.5	14.7	19.7
**Women, %**	61.8	58.9	54.1
**Education, %[d]**			
Low	52.7	50.7	21.8
Medium	41.9	43.5	6.7
High	5.4	5.9	71.6
**Annual household income, %[e]**			
Low	62.4	57.0	24.6
Medium	37.6	43.0	51.2
High			24.3
**Smoking %[f]**			
Never	64.4	61.8	54.2
Ex	5.3	6.0	34.4
Current	30.3	32.2	11.3
**Alcohol %[g]**			
Never	47.3	45.8	8.6
Ex	1.6	1.8	-
Current	51.2	52.4	91.4
**BMI (kg/m^2), mean (SD)**	23.7 (3.4)	23.7 (3.4)	27.6 (4.8)
**BMI (kg/m^2), %**			
<18.5	3.9	4.4	0.5
18.5-24.9	62.6	62.7	31.5
25.0-29.9	29.2	28.8	42.8
≥30	4.4	4.1	25.2
**Self-rated health status, %**			
Excellent	18.0	17.6	15.3
Good	29.5	28.1	57.6
Fair	42.7	44.0	22.1
Poor	9.8	10.4	4.9

[a]Baseline characteristics of CKB participants, who attended the second resurvey and were included in the analysis sample of the current study.

[b]Baseline characteristics of CKB participants, who did not attend the second resurvey and were not included in the analysis sample of the current study.

[c]Proportion of participants with missing values ranges from 0.6% (BMI) to 21% (education) in UKB. However, there are no missing values in CKB.

[d]Low level of education indicates primary school/no formal education in CKB and national examination (16 yr) for UKB. Medium level of education represents middle school or high school education in CKB and national examination (17/18 yr) for UKB. High level of education means college or university for both studies.

[e]Low level of income indicates <20,000 yuan/yr in CKB and <18,000 £/yr in UKB. Medium and high levels of income in CKB are presented as combined and indicate ≥20,000 yuan/yr in CKB, since at baseline the information for the highest levels of income was obtained in categories of 20,000-34,999 yuan/yr and ≥35,000 yuan/yr. Medium and high levels of income in UKB indicate 18,000-<52,000 £/yr and ≥52,000 £/yr, respectively.

[f]Within the CKB 25,040 sample there were 14.0 %, 12.8% and 73.2% men and 95.6%, 0.6% and 3.8% women, who were never smokers, ex-smokers, and current smokers, respectively. Within the CKB 487,486 sample, there were 14.4%, 13.3% and 72.3% men and 94.9%, 0.9% and 4.2% women, who were never smokers, ex-smokers, and current smokers, respectively. In the UKB sample, there were 48.4%, 38.3% and 13.4% men and 59.2%, 31.2% and 9.6% women, who were never smokers, ex-smokers, and current smokers, respectively.

[g]Within the CKB 25,040 sample there were 19.3%, 3.3% and 77.5% men and 64.6%, 0.5% and 34.9% women, who were never alcohol drinkers, ex-alcohol drinkers, and current alcohol drinkers, respectively. Within the CKB 486,487 sample, there were 20.4%, 3.8% and 75.8% men and 63.5%, 0.4% and 36.1% women, who were never alcohol drinkers, ex-alcohol drinkers, and current alcohol drinkers, respectively. Within the UKB sample, there were 6.7% and 93.3% men and 10.2% and 89.9% women, who were never alcohol drinkers and current alcohol drinkers, respectively.

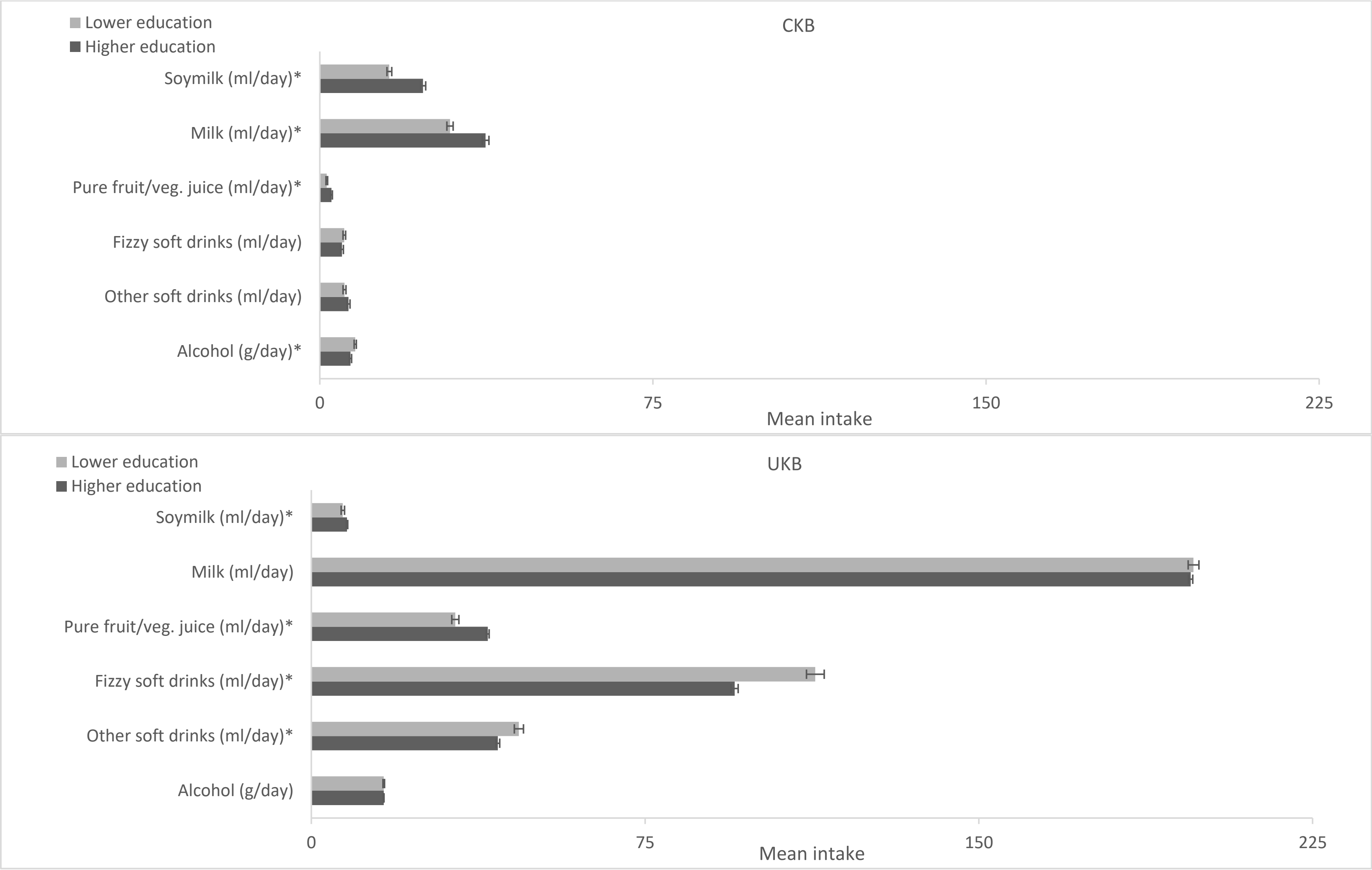

Figure S1: Adjusted[a] mean intake (ml or g/day) of drinks in participants with lower and higher levels (medium and high combined) of education in CKB and UKB

[a]Adjusted for age (<45, 45–49, 50–54, 55–59, 60–64, and ≥65 years), sex (men and women), region (10 relevant for each cohort) and income (lower [low and medium combined] and higher levels). More details on the definitions of lower and higher levels of education in each study can be found in Table 1. Phet <0.01 for fixed-effects meta-analysis between CKB and UKB low education levels and CKB and UKB high education levels. *P <0.05 for independent Student's T-test between CKB low and high education and UKB low and high education.

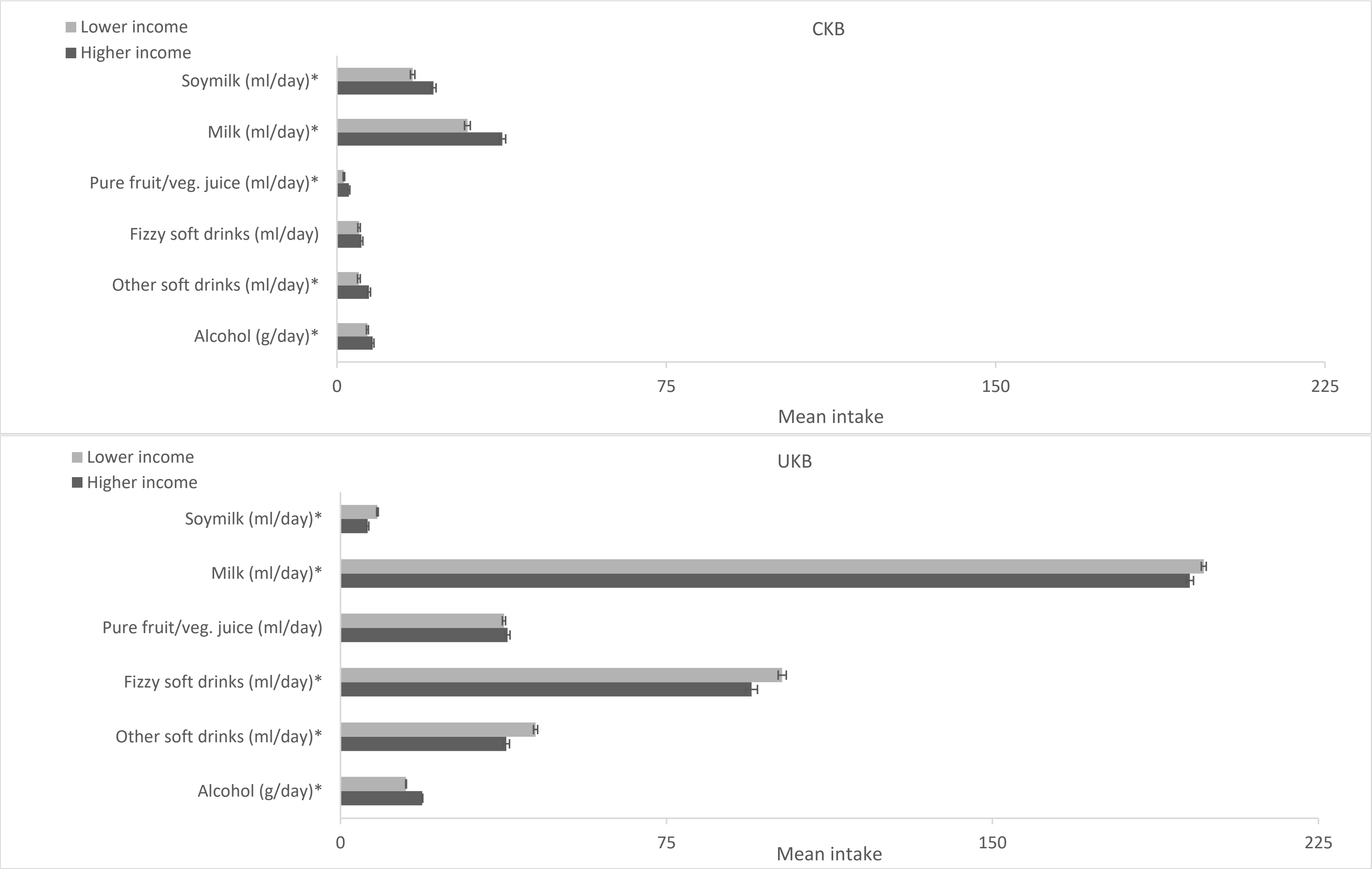

Figure S2: Adjusted[a] mean intake (ml or g/day) of drinks in participants with lower (low and medium combined) and higher levels of household income in CKB and UKB
[a]Adjusted for age (<45, 45–49, 50–54, 55–59, 60–64, and ≥65 years), sex (men and women), region (10 relevant for each cohort) and education (lower and higher levels [medium and high combined]). More details on the definitions of lower and higher levels of household income in each study can be found in Table 1. Phet <0.01 for fixed-effects meta-analysis between CKB and UKB low income levels and CKB and UKB high income levels.*P <0.05 for independent Student's T-test between CKB low and high income and UKB low and high income.

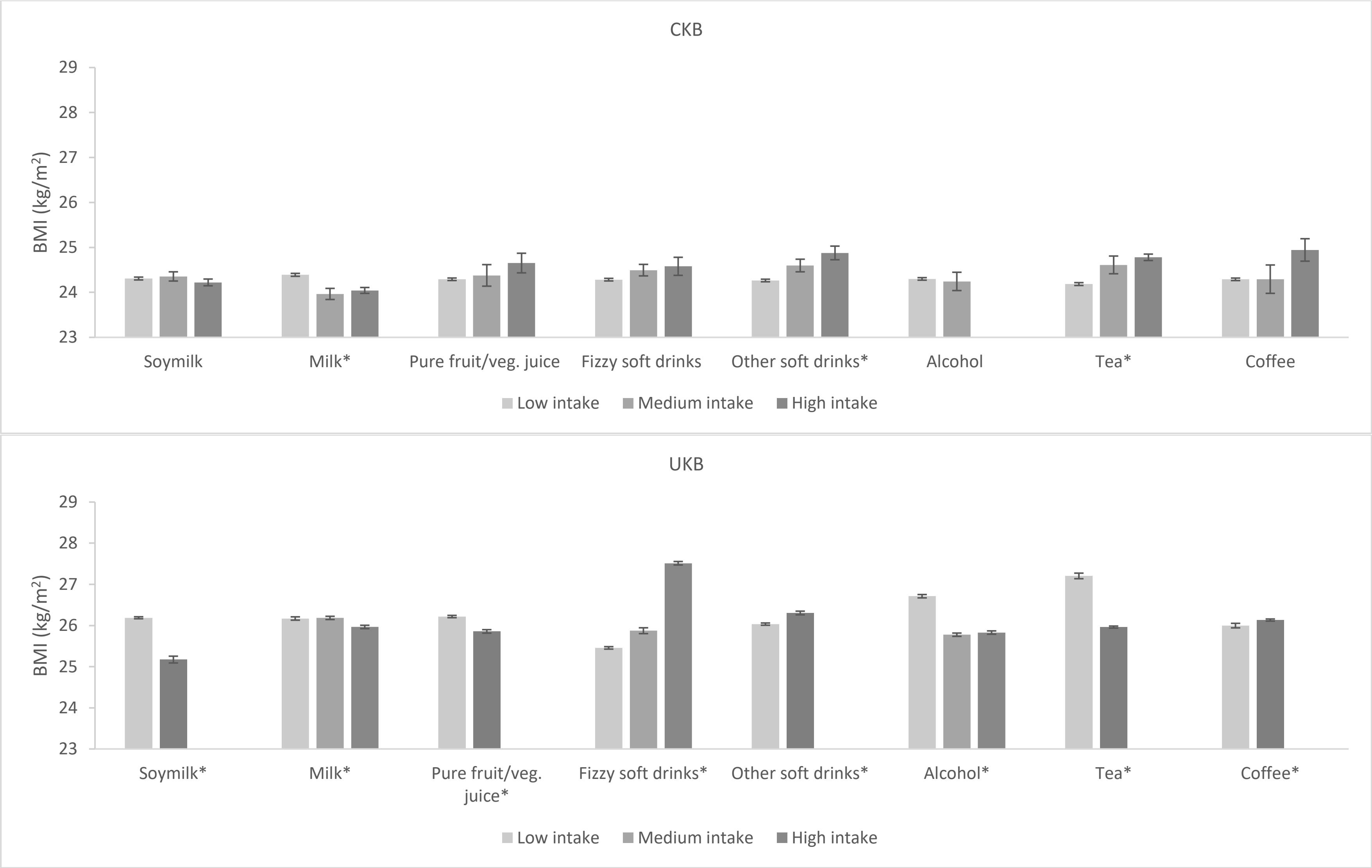

Figure S3. Adjusted[a] mean BMI by intake of drinks in CKB and UKB in women

[a]Adjusted for age (<45, 45–49, 50–54, 55–59, 60–64, and ≥65 years), region (10 relevant for each cohort), education level (low, medium, high), income level (low, medium, high), smoking (never, ex-smoker, current smoker), physical activity (quartiles of metabolic equivalent of task hours per day in CKB and quartiles of metabolic equivalent of task hours per week in UKB), and alcohol (never, ex-drinker, current drinker in CKB and never, current drinker in UKB except in the analyses with alcohol intake [g/day]). In CKB intake of drinks was divided into above and below median for alcohol (g/d), tertiles where possible or low, medium and high intake corresponding to never/rarely, monthly and weekly intake, respectively. In UKB intake of drinks was divided into tertiles where possible or 'none' versus 'any' where intakes were too low. *P-trend <0.05 across the intake groups.

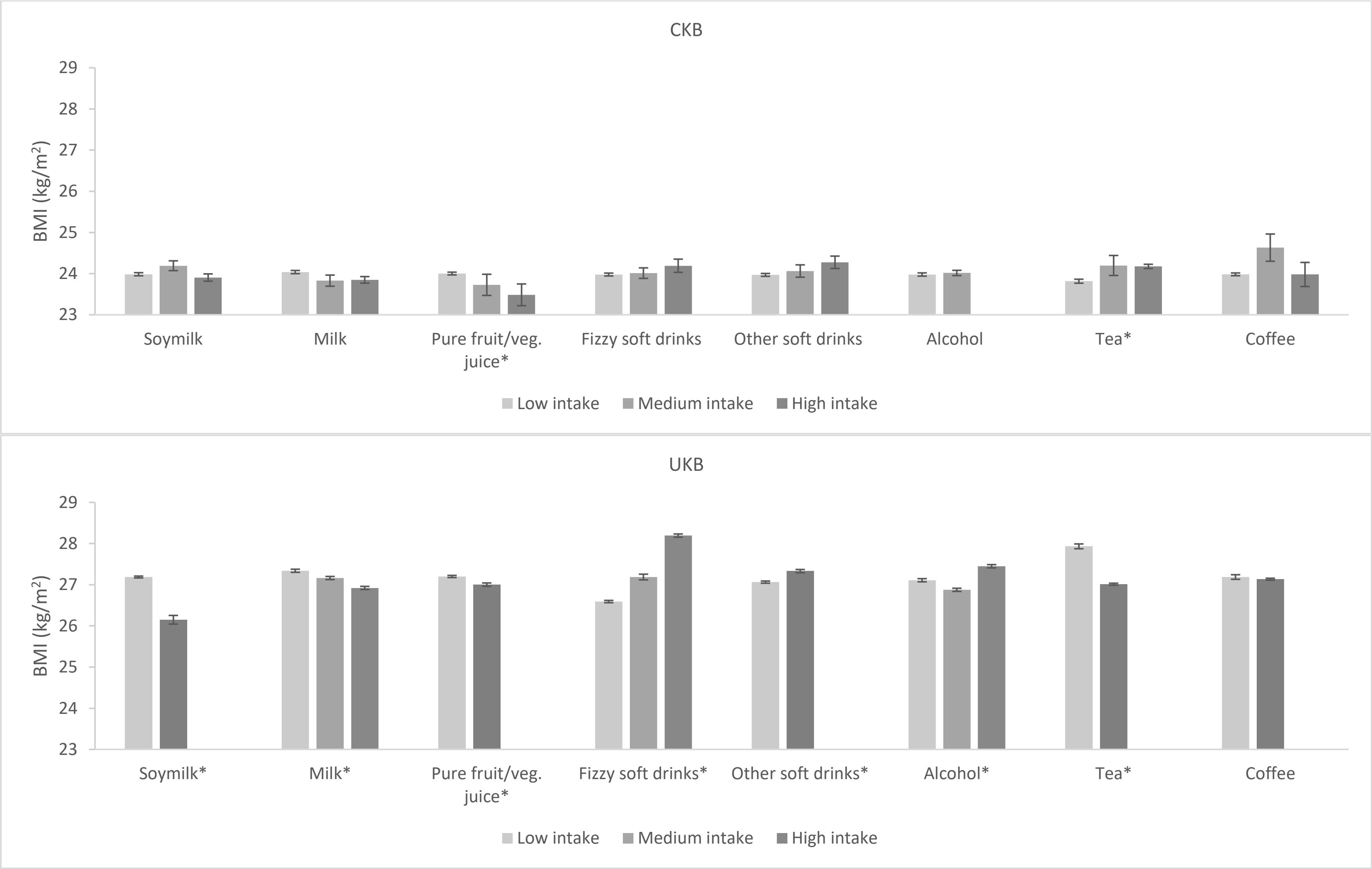

Figure S4. Adjusted[a] mean BMI by intake of drinks in CKB and UKB in men

[a]Adjusted for age (<45, 45–49, 50–54, 55–59, 60–64, and ≥65 years), region (10 relevant for each cohort), education level (low, medium, high), income level (low, medium, high), smoking (never, ex-smoker, current smoker), physical activity (quartiles of metabolic equivalent of task hours per day in CKB and quartiles of metabolic equivalent of task hours per week in UKB), and alcohol (never, ex-drinker, current drinker in CKB and never, current drinker in UKB except in the analyses with alcohol intake [g/day]). In CKB intake of drinks was divided into above and below median for alcohol (g/d), tertiles where possible or low, medium and high intake corresponding to never/rarely, monthly and weekly intake, respectively. In UKB intake of drinks was divided into tertiles where possible or 'none' versus 'any' where intakes were too low. *P-trend <0.05 across the intake groups.
